# Supplementary material for: Phosphorylation of PACSIN2 at S313 Regulates Podocyte Architecture in Coordination with N-WASP
Source: Cells. 2023 May 27;12(11):1487. doi: 10.3390/cells12111487 (PMC10252800; doi:10.3390/cells12111487)
Supplement: Supplementary file 1 [file cells-12-01487-s001.zip › cells-2177973-supplementary.pdf]

cells

MDPI

Supplementary Information for

Phosphorylation of PACSIN2 at S313 Regulates Podocyte Architecture in Coordination with N-WASP

Rim Bouslama [1,†], Vincent Dumont [1,†], Sonja Lindfors [1], Lassi Paavolainen [2], Jukka Tienari [3], Harry Nisen [4], Tuomas Mirtti [5,6], Moin A. Saleem [7], Daniel Gordin [8,9,10], Per-Henrik Groop [1,11,12,13], Shiro Suetsugu [14,15,16] and Sanna Lehtonen [1,17,*]

1 Research Program for Clinical and Molecular Metabolism, Faculty of Medicine, University of Helsinki, 00290 Helsinki, Finland
2 Institute for Molecular Medicine Finland (FIMM), Helsinki Institute of Life Science (HiLIFE), University of Helsinki, 00290 Helsinki, Finland
3 Department of Pathology, University of Helsinki, Helsinki, and Helsinki University Hospital, 05850 Hyvinkää, Finland
4 Department of Urology, Helsinki University Hospital, 00029 HUS, Finland; nisehar@gmail.com
5 Department of Pathology, Helsinki University Hospital, 00290 Helsinki, Finland
6 Research Program in Systems Oncology, Faculty of Medicine, University of Helsinki, 00290 Helsinki, Finland
7 Children's Renal Unit, Bristol Medical School, University of Bristol, Bristol BS8 1TS, UK
8 Minerva Foundation Institute for Medical Research, 00290 Helsinki, Finland
9 Abdominal Center, Nephrology, University of Helsinki and Helsinki University Hospital, 00290 Helsinki, Finland
10 Joslin Diabetes Center, Harvard Medical School, Boston, MA 02215, USA
11 Folkhälsan Institute of Genetics, Folkhälsan Research Center, 00290 Helsinki, Finland
12 Department of Nephrology, University of Helsinki and Helsinki University Hospital, 00290 Helsinki, Finland
13 Department of Diabetes, Central Clinical School, Monash University, Melbourne, VIC 3800, Australia
14 Division of Biological Science, Graduate School of Science and Technology, Nara Institute of Science and Technology, Ikoma 630-0192, Japan
15 Data Science Center, Nara Institute of Science and Technology, Ikoma 630-0192, Japan
16 Center for Digital Green-Innovation, Nara Institute of Science and Technology, Ikoma 630-0192, Japan
17 Department of Pathology, University of Helsinki, 00290 Helsinki, Finland
* Correspondence: sanna.h.lehtonen@helsinki.fi; Tel.: +358-50-4482798
† These authors contributed equally to this work.

This document file includes:

Supplementary Materials: Sequencing results of the overexpression constructs
Tables S1 to S5
Figure S1 to S4

Sequencing results of the overexpression constructs

Notes:

ATG (met) for both myc and PACSIN2, as well as TGA (stop) are in bold.

The nucleotides that differ between the wt, S313E and S313A constructs are underlined.

Nucleotides coding myc are shown in italic. The remaining nucleotides from the multiple cloning site are in smaller font.

myc-PACSIN2-wt sequence:

ATG*GCATCAATGCAGAAGCTGATCTCAGAGGAGGACCTG*CTTATGGCCATGGAGGCCCGAATTCTA**ATG**TCT
GTCACCTACGATGACTCTGTGGGAGTGGAAGTGTCCAGCGACAGCTTCTGGGAGGTTGGGAAC
TACAAACGGACTGTGAAGCGGATTGACGATGGCCACCGCCTGTGTGGTGACCTCATGAACTGT
CTGCATGAGCGGGCACGCATCGAGAAGGCGTATGCACAGCAGCTCACTGAGTGGGCCCGACGC
TGGAGGCAGCTGGTAGAGAAGGGACCACAGTATGGGACCGTGGAGAAGGCCTGGATAGCTGT
CATGTCTGAAGCAGAGAGGGTGAGTGAACTGCACCTGGAAGTGAAGGCATCACTGATGAATG
AAGACTTTGAGAAGATCAAGAACTGGCAGAAGGAAGCCTTTCACAAGCAGATGATGGGAGGC
TTCAAGGAGACCAAAGAAGCAGAGGATGGCTTTCGGAAGGCCCAGAAGCCCTGGGCCAAGAA
GCTGAAAGAGGTGGAAGCGGCAAAGAAGGCGCACCACACAGCGTGCAAAGAGGAGAAGCTG
GCCATCTCCCGGGAAGCCAACAGCAAGGCAGATCCATCCCTCAACCCTGAGCAGCTGAAGAAA
CTGCAAGACAAGATAGAAAAATGCAAACAGGATGTTCTAAAGACCAAGGACAAGTATGAGAA
GTCCCTGAAGGAGCTTGATCAGACCACACCCCAGTACATGGAGAACATGGAGCAGGTGTTCGA
GCAGTGCCAGCAGTTTGAAGAGAAGCGCCTGCGCTTCTTCCGGGAGGTTCTGCTGGAGGTTCA
GAAGCACTTGGATCTGTCCAATGTGGCTAGCTATAAAACCATTTACCGGGAGCTGGAGCAGAG
CATCAAAGCAGCAGATGCGGTAGAGGACCTGAGGTGGTTCCGGGCTAACCATGGGCCAGGCAT
GGCTATGAACTGGCCACAGTTTGAGGAGTGGTCTGCAGATCTGAATCGAACTCTCAGCCGGAG
AGAGAAGAAGAAGGCTGTTGACGGTGTCACCCTAACAGGGATCAACCAGACAGGTGACCAGT
CTGGACAGAACAAGCCTGGCAGCAACCTTAGTGTCCCGAGCAACCCCGCCCAGTCCACGCAGT
TACAGTCCAGCTACAACCCCTTCGAGGACGAGGACGACACGGGCAGCAGCATCAGTGAGAAG
GAGGACATTAAGGCCAAAAATGTCAGCAGCTATGAGAAGACTCAGACTTACCCCACTGACTGG
TCTGATGATGAGTCTAACAACCCTTTCTCCTCCACGGATGCCAACGGGGATTCGAACCCATTTG
ATGAGGACACGACCTCAGGAACAGAAGTGCGAGTTCGGGCCCTCTATGACTATGAGGGGCAG
GAACATGATGAGCTGAGCTTCAAGGCTGGGGATGAACTGACCAAGATAGAGGATGAAGATGA

ACAGGGTTGGTGCAAGGGACGTTTAGACAGCGGCCAGGTTGGCCTATACCCAGCCAACTATGT
CGAGGCTATCCAG**TGA**

myc-PACSIN2-S313E sequence:

ATG*GCATCAATGCAGAAGCTGATCTCAGAGGAGGACCTG*CTTATGGCCATGGAGGCCCGAATTCTA**ATG**T
CTGTCACCTACGATGACTCTGTGGGAGTGGAAGTGTCCAGCGACAGCTTCTGGGAGGTTGGGA
ACTACAAACGGACTGTGAAGCGGATTGACGATGGCCACCGCCTGTGTGGTGACCTCATGAACT
GTCTGCATGAGCGGGCACGCATCGAGAAGGCGTATGCACAGCAGCTCACTGAGTGGGCCCGAC
GCTGGAGGCAGCTGGTAGAGAAGGGACCACAGTATGGGACCGTGGAGAAGGCCTGGATAGCT
GTCATGTCTGAAGCAGAGAGGGTGAGTGAACTGCACCTGGAAGTGAAGGCATCACTGATGAAT
GAAGACTTTGAGAAGATCAAGAACTGGCAGAAGGAAGCCTTTCACAAGCAGATGATGGGAGG
CTTCAAGGAGACCAAAGAAGCAGAGGATGGCTTTCGGAAGGCCCAGAAGCCCTGGGCCAAGA
AGCTGAAAGAGGTGGAAGCGGCAAAGAAGGCGCACCACACAGCGTGCAAAGAGGAGAAGCT
GGCCATCTCCCGGGAAGCCAACAGCAAGGCAGATCCATCCCTCAACCCTGAGCAGCTGAAGAA
ACTGCAAGACAAGATAGAAAAATGCAAACAGGATGTTCTAAAGACCAAGGACAAGTATGAGA
AGTCCCTGAAGGAGCTTGATCAGACCACACCCCAGTACATGGAGAACATGGAGCAGGTGTTCG
AGCAGTGCCAGCAGTTTGAAGAGAAGCGCCTGCGCTTCTTCCGGGAGGTTCTGCTGGAGGTTC
AGAAGCACTTGGATCTGTCCAATGTGGCTAGCTATAAAACCATTTACCGGGAGCTGGAGCAGA
GCATCAAAGCAGCAGATGCGGTAGAGGACCTGAGGTGGTTCCGGGCTAACCATGGGCCAGGC
ATGGCTATGAACTGGCCACAGTTTGAGGAGTGGTCTGCAGATCTGAATCGAACTCTCGAGCGG
AGAGAGAAGAAGAAGGCTGTTGACGGTGTCACCCTAACAGGGATCAACCAGACAGGTGACCA
GTCTGGACAGAACAAGCCTGGCAGCAACCTTAGTGTCCCGAGCAACCCCGCCCAGTCCACGCA
GTTACAGTCCAGCTACAACCCCTTCGAGGACGAGGACGACACGGGCAGCAGCATCAGTGAGA
AGGAGGACATTAAGGCCAAAAATGTCAGCAGCTATGAGAAGACTCAGACTTACCCCACTGACT
GGTCTGATGATGAGTCTAACAACCCTTTCTCCTCCACGGATGCCAACGGGGATTCGAACCCATT
TGATGAGGACACGACCTCAGGAACAGAAGTGCGAGTTCGGGCCCTCTATGACTATGAGGGGCA
GGAACATGATGAGCTGAGCTTCAAGGCTGGGGATGAACTGACCAAGATAGAGGATGAAGATG
AACAGGGTTGGTGCAAGGGACGTTTAGACAGCGGCCAGGTTGGCCTATACCCAGCCAACTATG
TCGAGGCTATCCAG**TGA**

myc-PACSIN2-S313A sequence:

ATG*GCATCAATGCAGAAGCTGATCTCAGAGGAGGACCTG*CTTATGGCCATGGAGGCCCGAATTCTA**ATG**T
CTGTCACCTACGATGACTCTGTGGGAGTGGAAGTGTCCAGCGACAGCTTCTGGGAGGTTGGGA
ACTACAAACGGACTGTGAAGCGGATTGACGATGGCCACCGCCTGTGTGGTGACCTCATGAACT
GTCTGCATGAGCGGGCACGCATCGAGAAGGCGTATGCACAGCAGCTCACTGAGTGGGCCCGAC

GCTGGAGGCAGCTGGTAGAGAAGGGACCACAGTATGGGACCGTGGAGAAGGCCTGGATAGCT
GTCATGTCTGAAGCAGAGAGGGTGAGTGAACTGCACCTGGAAGTGAAGGCATCACTGATGAAT
GAAGACTTTGAGAAGATCAAGAACTGGCAGAAGGAAGCCTTTCACAAGCAGATGATGGGAGG
CTTCAAGGAGACCAAAGAAGCAGAGGATGGCTTTCGGAAGGCCCAGAAGCCCTGGGCCAAGA
AGCTGAAAGAGGTGGAAGCGGCAAAGAAGGCGCACCACACAGCGTGCAAAGAGGAGAAGCT
GGCCATCTCCCGGGAAGCCAACAGCAAGGCAGATCCATCCCTCAACCCTGAGCAGCTGAAGAA
ACTGCAAGACAAGATAGAAAAATGCAAACAGGATGTTCTAAAGACCAAGGACAAGTATGAGA
AGTCCCTGAAGGAGCTTGATCAGACCACACCCCAGTACATGGAGAACATGGAGCAGGTGTTCG
AGCAGTGCCAGCAGTTTGAAGAGAAGCGCCTGCGCTTCTTCCGGGAGGTTCTGCTGGAGGTTC
AGAAGCACTTGGATCTGTCCAATGTGGCTAGCTATAAAACCATTTACCGGGAGCTGGAGCAGA
GCATCAAAGCAGCAGATGCGGTAGAGGACCTGAGGTGGTTCCGGGCTAACCATGGGCCAGGC
ATGGCTATGAACTGGCCACAGTTTGAGGAGTGGTCTGCAGATCTGAATCGAACTCTCGCCCGG
AGAGAGAAGAAGAAGGCTGTTGACGGTGTCACCCTAACAGGGATCAACCAGACAGGTGACCA
GTCTGGACAGAACAAGCCTGGCAGCAACCTTAGTGTCCCGAGCAACCCCGCCCAGTCCACGCA
GTTACAGTCCAGCTACAACCCCTTCGAGGACGAGGACGACACGGGCAGCAGCATCAGTGAGA
AGGAGGACATTAAGGCCAAAAATGTCAGCAGCTATGAGAAGACTCAGACTTACCCCACTGACT
GGTCTGATGATGAGTCTAACAACCCTTTCTCCTCCACGGATGCCAACGGGGATTCGAACCCATT
TGATGAGGACACGACCTCAGGAACAGAAGTGCGAGTTCGGGCCCTCTATGACTATGAGGGGCA
GGAACATGATGAGCTGAGCTTCAAGGCTGGGGATGAACTGACCAAGATAGAGGATGAAGATG
AACAGGGTTGGTGCAAGGGACGTTTAGACAGCGGCCAGGTTGGCCTATACCCAGCCAACTATG
TCGAGGCTATCCAG**TGA**

Table S1. Characteristics of the individuals with T2D and people without diabetes whose glomeruli were used to define the expression level of total PACSIN2 and pS313-PACSIN2 in figure 2.

	**Gender (m/f)**	**Age (y)**	**BMI (kg/m²)**	**Hypertens. med.**	**Hyperchol. med.**
Ctrl	6/5	65.7±8.9	26.3±4.5	5/11	3/11
T2D	8/2	74.4±9.5	28.9±5.5	9/10	4/10

* Hypertens. med.: medication for hypertension, hyperchol. med.: medication for hypercholesterolemia

Table S2. Characteristics of the individuals with T2D whose sera were used to stimulate podocytes in figure 2.

	**Age (y)**	**BMI (kg/m²)**	**Hypertens. med.**	**Hyperchol. med.**	**Glucose (mmol/L)**	**HBA1c (%)**	**Chol. (mmol/dL)**	**HDL (mmol/dL)**	**Trigly. (mmol/dL)**
Normoalb.	66.0±2.2	32.3±7.6	8/10	8/10	6.22±2.08	7.26±0.96	4.01±0.82	1.56±0.44	1.09±0.33
Microalb.	61.0±5.1	34.9±4.3	9/10	8/10	7.08±0.92	7.79±1.58	4.17±0.65	1.27±0.38	1.70±1.06

* Normoalb.: normoalbuminuria, Microalb.: microalbuminuria, Hypertens. med.: medication for hypertension, Hyperchol. med.: medication for hypercholesterolemia, HBA1c: glycated haemoglobin A1c, Chol.: cholesterol, HDL: high-density lipoproteins, Trigly.: triglycerides

Table S3. Descriptive statistics for cell size analysis Figure 5G.

	**GFP-EV**		**GFP-WT**		**GFP-S313E**		**GFP-S313A**	
	DMSO	wiskostatin	DMSO	wiskostatin	DMSO	wiskostatin	DMSO	wiskostatin
Number of values	198	147	28	52	110	31	69	134
Minimum	207.1	206.9	262.6	686.2	217.3	755.2	551.9	151.5
Maximum	10305	58183	29537	50628	146870	54069	50392	60879
Range	10098	57976	29274	49942	146653	53314	49840	60727
Mean	2747	2550	10135	9247	7905	9485	8445	4381
Std. Deviation	1694	5425	8974	9675	16468	11189	9954	7326
Std. Error of Mean	120.4	447.4	1696	1342	1570	2010	1198	632.9

Table S4. Descriptive statistics for cell size analysis Figure 7B.

	GFP-EV	GFP-WT	GFP-S313E	GFP-S313A
Number of values	1098	436	895	901
Minimum	0.000	0.000	0.000	0.000
Maximum	423.0	935.0	625.0	710.0
Range	423.0	935.0	625.0	710.0
Mean	62.18	98.55	70.61	70.16
Std. Deviation	44.11	81.87	56.57	56.42
Std. Error of Mean	1.331	3.921	1.891	1.879
Lower 95% CI of mean	59.57	90.84	66.90	66.48
Upper 95% CI of mean	64.79	106.3	74.32	73.85

Table S5. Descriptive statistics for focal adhesion number analysis Figure 7D.

	GFP-EV	GFP-WT	GFP-S313E	GFP-S313A
Number of values	1098	436	895	901
Minimum	0.000	0.000	0.000	0.000
Maximum	423.0	935.0	625.0	710.0
Range	423.0	935.0	625.0	710.0
Mean	62.18	98.55	70.61	70.16
Std. Deviation	44.11	81.87	56.57	56.42
Std. Error of Mean	1.331	3.921	1.891	1.879
Lower 95% CI of mean	59.57	90.84	66.90	66.48
Upper 95% CI of mean	64.79	106.3	74.32	73.85

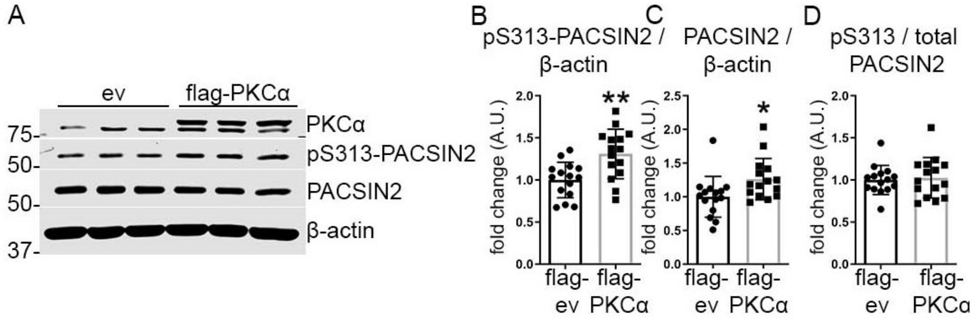

Figure S1. PKCα regulates PACSIN2 expression and phosphorylation. **(A-D)** Quantification of Western blots of total PACSIN2, pS313-PACSIN2 (A) in lysates from proliferating human podocytes transiently overexpressing flag-ev or flag-PKCα shows increased expression of PACSIN2 (C) and phosphorylation at S313 (B) in podocytes overexpressing flag-PKCα to ev control. β-actin is used as a loading control. n=15 from 5 independent experiments in B, n=9 from 3 independent experiments in C-E, and n=15 from 5 independent experiments. *: p<0.05, **: p<0.01 vs ev.

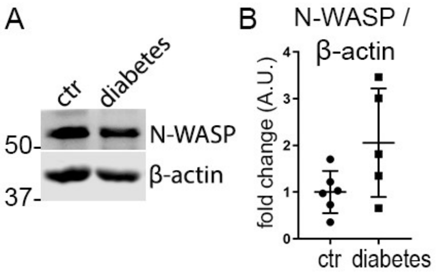

Figure S2. N-WASP tends to increase in the glomeruli of individuals with T2D. **(A,B)** Quantification of Western blots of N-WASP (A) in lysates of glomeruli isolated from individuals with T2D shows a trend of increase in the expression N-WASP normalized to β-actin in individuals with T2D compared to controls (ctr) (B). n=6 in ctr and 6 in diabetes.

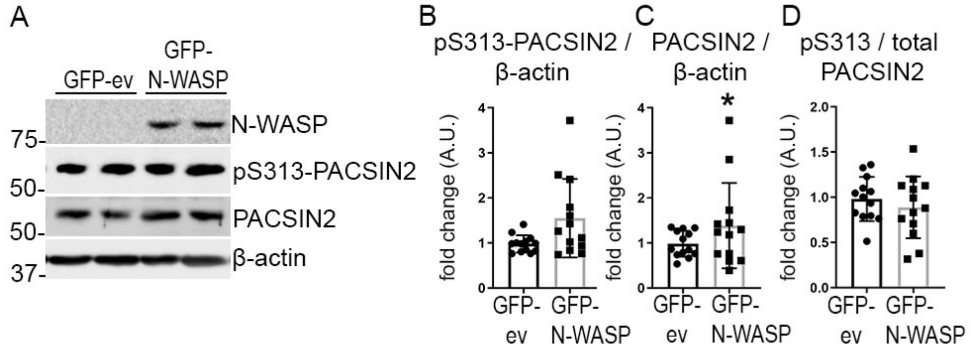

Figure S3. N-WASP regulates PACSIN2. **(A-D)** Quantification of Western blots of total PACSIN2 and pS313-PACSIN2 (A) in lysates from proliferating human podocytes transiently overexpressing GFP-empty vector (GFP-ev) or GFP-N-WASP shows increased expression of PACSIN2 (C) in podocytes overexpressing GFP-N-WASP in comparison to empty vector control. β-actin is used as a loading control. n=13 from 4 independent experiments *: p<0.05 vs ev.

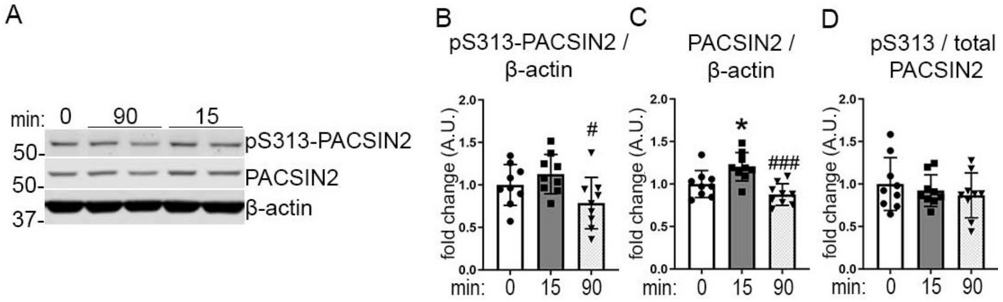

Figure S4. PACSIN2 expression and phosphorylation vary during the recovery from cytochalasin D. **(A-D)** Quantification of Western blots of total PACSIN2 and pS313-PACSIN2 (A) in lysates from differentiated human podocytes after treatment with cytochalasin D and washout showing that expression of PACSIN2 increases 15 min after washout and decreases after 90 min (C), whereas as pS313-PACSIN2 decreases after 90 min (A). β-actin is used as a loading control. n=9 from 3 independent experiments. ** vs 0 min. # vs 15 min. *: p<0.05, #: p<0.05, ###: p<0.001.
